# On the Application of Galvanism

**Published:** 1802-12-01

**Authors:** Richard Teed

**Affiliations:** Lancaster Court, Strand


### *To the Editors of the Medical and Phyfical Journal.*

Gentlemen,

In the month of March laft, I communicated to the public, through the medium of the Philofophical Magazine, the refult of an experiment made with a belt or chain on the principle of a Galvanic pile, and applied with fingular fuccefs in a cafe of rheumatifm, with which I had been troubled for near a year, and which had, until I ufed the belt, increafed to an alarming degree. I have the pleafure to inform your readers, who might have fimilar complaints in the back and loins, and who may be inclined to make trial of the belt, which is compofed of plates of zinc, united by links of plated copper wire *; as alfo for the fatisfaction of thofe who have made enquiries, that I ftill wear it; nor do I mean to leave it off, being well convinced from the experiment alluded to above, and having now worn it a year, that it has fo far accomplifhed my wifhes in the removal of my complaint, as enables me to affure others, that I have not felt the leaft return for five minutes fince I have applied the belt a fecond time. In making this known, I have no other motive but to leffen in a fmall degree, or relieve the fufferings of mankind: and although the fame fuccefs may not attend others with complaints fomewhat refembling mine; as, for inftance, the gravel, where I have known it has failed, I am neverthelefs perfuaded, this newly difcovered Galvanic fluid will only require to be differently modified in its application to the human

* See Philofophical Magazine, March 1802.

human, and perhaps the brutal frame, in order to reſtore that equilibrium of the electric fluid ſo neceſſary to health, without which neither the animal nor vegetable world could long ſubſiſt. I believe it is now pretty well underſtood, that this is only another branch of electricity, and we know that electricity has been of conſiderable benefit when properly applied in certain diſorders, but not ſo in all; this might be the caſe with the Galvanic fluid; and we have every reaſon to hope at leaſt, that it might, by enlarging or multiplying the zinc plates, effect a removal of the complaint, where the old or common electricity has failed. In the new electricity there is moſt certainly this conſiderable advantage, that the Galvanic belt is an electrical apparatus, conſtantly in action as long as it is worn, and I believe more powerful when perſpiration is promoted; I therefore could wiſh ſcientific men would treat the ſubject like philoſophers, and not be influenced by *prejudice*, that great obſtacle to very important diſcoveries.

I am, &c.

*Lancaster Court, Strand,*
17 *Sept.* 1802.

RICHARD TEED.

